# Understanding changes in the hydrological behaviour within a karst aquifer (Lurbach system, Austria)

**DOI:** 10.1007/s13146-013-0172-3

**Published:** 2013-10-04

**Authors:** Cyril Mayaud, Thomas Wagner, Ralf Benischke, Steffen Birk

**Affiliations:** 1 Institute for Earth Sciences, University of Graz, Heinrichstraße 26, 8010 Graz, Austria; 2 Department of Water Resources and Environmental Analytics, Institute for Water, Energy and Sustainability, Joanneum Research Forschungsgesellschaft mbH, Elisabethstraße 18/II, 8010 Graz, Austria

**Keywords:** Binary karst aquifer, MODFLOW, Single-continuum model, Groundwater modelling, Laminar/turbulent flow, Hydrological behaviour

## Abstract

A thorough data analysis combined with groundwater modelling was conducted in an Austrian binary karst aquifer to better understand changes in the hydrological behaviour observed at a karst spring. During a period of 4 years after a major flood event the spring hydrograph appears to be more damped with lower peak flow and higher baseflow than in the years before. The analysis of the hydrograph recession suggests that the observed hydrological change is caused by changes within the karst system rather than by varying hydro-meteorological conditions. The functioning of the aquifer and potential causes of the observed changes are further examined using the groundwater flow model MODFLOW. The simulation results suggest that a modification of hydraulic conductivity and storage within the conduit network, e.g. due to the plugging of the drainage conduits with sediments, may be the cause of the different behaviour. MODFLOW was able to reproduce the observed dynamics of spring flow, although it does not account for turbulent flow within karst conduits. Using a simplified model scenario it is demonstrated that the damping of the hydrograph is much stronger if turbulent conduit flow is taken into account. Thus, a turbulent flow model is needed to assess potential changes in the storage properties quantitatively.

## Introduction

Karst waters represent an important part of the water supply for the world’s population (20–25 %; Ford and Williams 2007), but are known for their high vulnerability to chemical and bacterial contamination (e.g. Heinz et al. 2009) due to the pressure of urbanization and intensive agricultural use. To assess how changes within karst areas might influence the behaviour of karst springs a sound understanding of processes which govern flow through karst aquifers is needed. This work examines the hydrological behaviour of the Hammerbach karst spring in Austria, which appears to have changed after a storm event in August 2005. The purpose here is to improve the general understanding of flow processes within karst aquifers and especially to identify potential causes of the observed change within this spring catchment. To this end, hydrograph data from the karst spring are analysed and interpreted based on the existing knowledge from earlier investigations to develop a simplified conceptual aquifer model. The functioning of the aquifer and potential causes of the different changes in the hydrological behaviour are further examined using a process-based numerical groundwater flow model.

## Field site

**Fig. 1:**
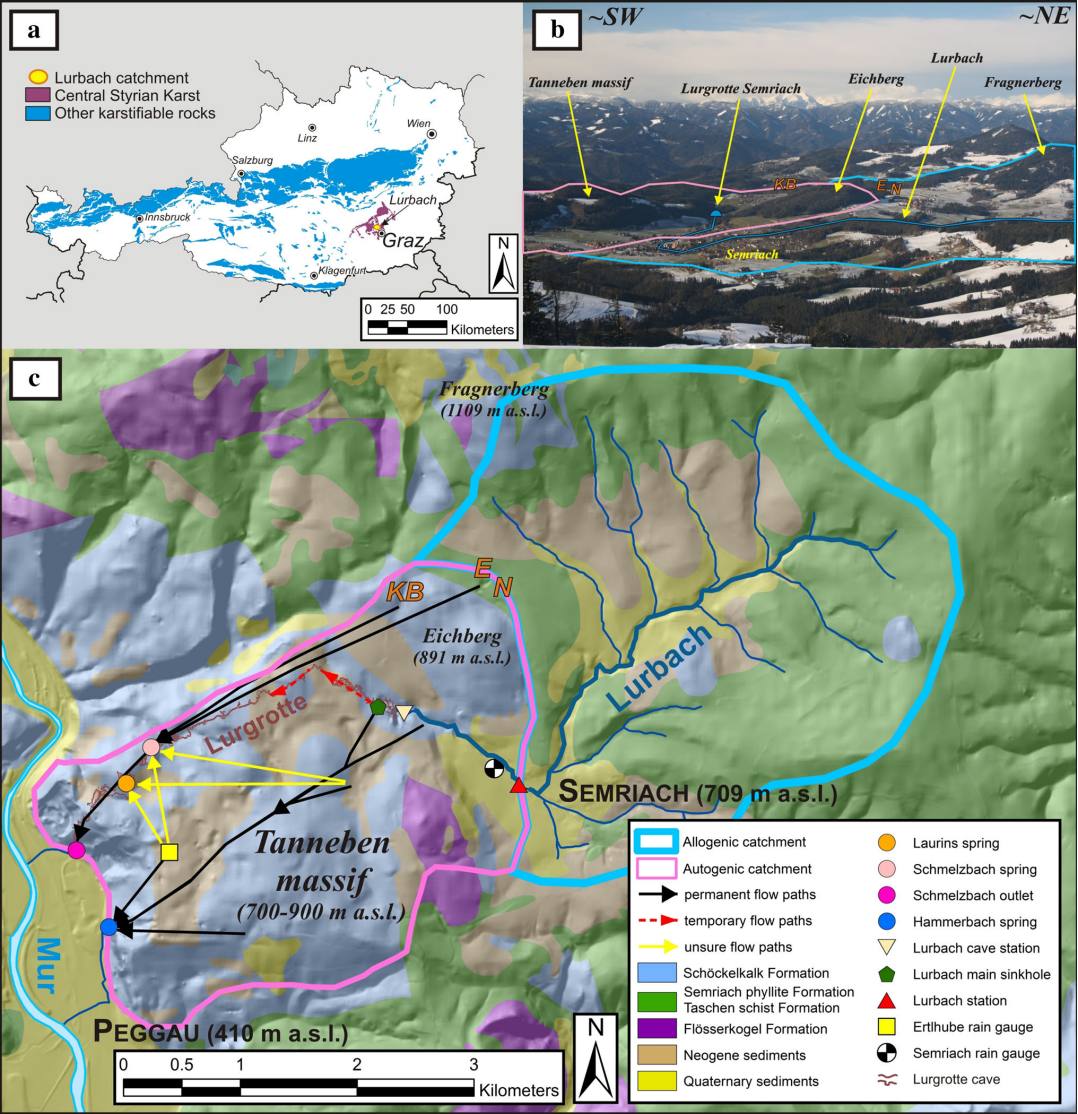
Field site location. **a** Map showing the location of the Lurbach system in relation to the distribution of karst rocks in Austria (modified after Schubert 2003). **b** View of the upper Lurbach catchment and the karstified area (the *blue and pink polygons*, respectively) taken from the summit of the Schöckl mountain (1,445 m a.s.l.). Photo: M. Schneider and C. Aistleitner (used with permission). **c** Simplified geological map (modified after Geologische Bundesanstalt 2005; Blatt 164-Graz) of the Lurbach catchment including the different subsurface flow paths (*black*, *red* and *yellow arrows*) inferred from results of tracer tests (the unsure flow paths were determined during a single tracer experiment with injection on top of the unsaturated zone and small recovery rates; Behrens et al. 1992), the measurement network and three minor sinkholes supplying the Schmelzbach spring (*E* Eisgrube, *N* Neudorferschwinde and *KB* Katzenbachschwinde, respectively). The low permeable part (*blue polygon*) corresponds to the topographic catchment, whereas the highly karstified part (*pink polygon*) was delineated taking into account results of tracers experiments. The boundary between the allogenic and the autogenic unit is based on the geological map

The investigation area is a binary karst system of 23 km^2^ named Lurbach system and located in the Central Styrian Karst (Fig. 1a), about 20 km north of Graz (Styria, Austria). The upper part of the catchment is a 15 km^2^ wide area of low permeable rocks (comprising mainly Quaternary sediments and Paleozoic schists), and is drained by the Lurbach stream in an East–West direction towards the Tanneben massif, a highly karstified limestone block with a sub-catchment size of 8 km^2^. After the Lurbach stream reaches the limestone unit, it infiltrates along the streambed and finally disappears into a major sinkhole located some tens of meters behind the entrance of a big cave, the Lurgrotte (located at 633 m a.s.l.). Then, the water flows through the fissures and conduits of the limestone massif and resurges at the Schmelzbach outlet and the Hammerbach spring, which are both located at the western border of the catchment, at the foot of a 300 m high cliff called Peggauer Wand. The highest point within the catchment is the Fragnerberg in the North (1,109 m a.s.l.; Fig. 1b) and the lowest is at the level of the Mur River (~400 m a.s.l.) at its western edge where the Hammerbach spring originates. The town of Semriach (709 m a.s.l.) is the main settlement in the area.

The Lurbach system is equipped with a measurement network well adapted to understand the karst processes. The discharge is measured at the Hammerbach spring and Schmelzbach outlet as well as the Lurbach prior to its disappearance into the ground: one gauge is located at the contact between the schists and the limestone and another one just in front of the Lurgrotte cave (Fig. 1c). However, not all of the measurement devices are operative at all times, mainly due to maintenance after flood events or general financial restrictions. As the Hammerbach discharge data are available on a continuous basis, it will be the focus of the modelling attempts presented below.

Six rain gauges are located in the vicinity of the catchment and provide rainfall data (only the Ertlhube and Semriach rain gauges are visible in Fig. 1c). Temperature data are collected at a meteorological station located close to the Lurbach system, on the western side of the Mur valley (at an altitude of 610 m a.s.l.). Unfortunately, no water table data are available within the limestone massif. Moreover, the conduit system draining towards the Hammerbach is not yet explored, as all attempts to access it failed up to the present. However, the Lurgrotte cave itself is a well explored partly water-active multi-level cave (e.g. Wagner et al. 2011) and shows relevant indications for the vadose/phreatic conditions inside parts of the Tanneben massif (Fig. 1c). Besides this, there are numerous other caves known in this region (more than 200 in the Tanneben massif). Yet their extents are rather small and they are generally plugged with sediments or collapse material preventing further exploration inside of the karst massif.

The Lurbach system is under a climate regime with low winter precipitation and is subject to heavy thunderstorm events during the summer (Harum and Stadler 1992). The mean annual precipitation measured between 1965 and 2011 is 880 mm. The maximum precipitation value recorded in one day was 93.5 mm during the summer of 1975.

The subsurface drainage pattern of the Lurbach system changes depending on the hydrological conditions (Harum and Stadler 1992):

1. *At low water conditions*, the Lurbach discharge becomes very small and sometimes intermittent (the minimum discharge measured at the Lurbach station is ~5–10 l/s) along the streambed between the upstream gauge of the Lurbach and the cave entrance (some sinkholes become visible along the stream bank). Then, the Hammerbach and Schmelzbach systems are totally separated, significantly fed by infiltration of the autogenic waters of the Tanneben massif and by a small cave-spring called Laurins spring (see in Fig. 1c) for the Schmelzbach.
2. *At normal water conditions*, the Lurbach disappears into the Lurgrotte and resurges mainly at the Hammerbach spring, whereas the Schmelzbach is only supplied by the autogenic recharge through the Tanneben massif, by the Laurins spring, and by three small sinkholes (E, N and KB; Fig. 1b, c) located in the NE boundary of the karstified area.
3. *At medium-to-high water conditions*, an overflow from the Hammerbach system to the Schmelzbach system is observed. Harum and Stadler (1992) showed that when the Hammerbach spring discharge increases to more than ~200 l/s, a part of the Lurbach water flows towards the Schmelzbach system.
4. *At flood conditions*, the Lurbach is subject to catastrophic flood events (the maximum discharge measured at the Lurbach station exceeded 10 m^3^/s) and the Lurgrotte cave system itself acts as the main drainage system (Fig. 1c). Then the Hammerbach system cannot drain more than ~2 m^3^/s, whereas the Schmelzbach outlet receives the most part of the Lurbach water and can reach peak discharges up to 10 m^3^/s and more. These flash floods caused sometimes the outage of measurement devices at the Schmelzbach outlet and within the active accessible cave stream. Then, redistribution of sediments and plugging of sinkholes and cave passages are regularly reported (Harum and Stadler 1992, p. 39).

## Data analysis

**Fig. 2:**
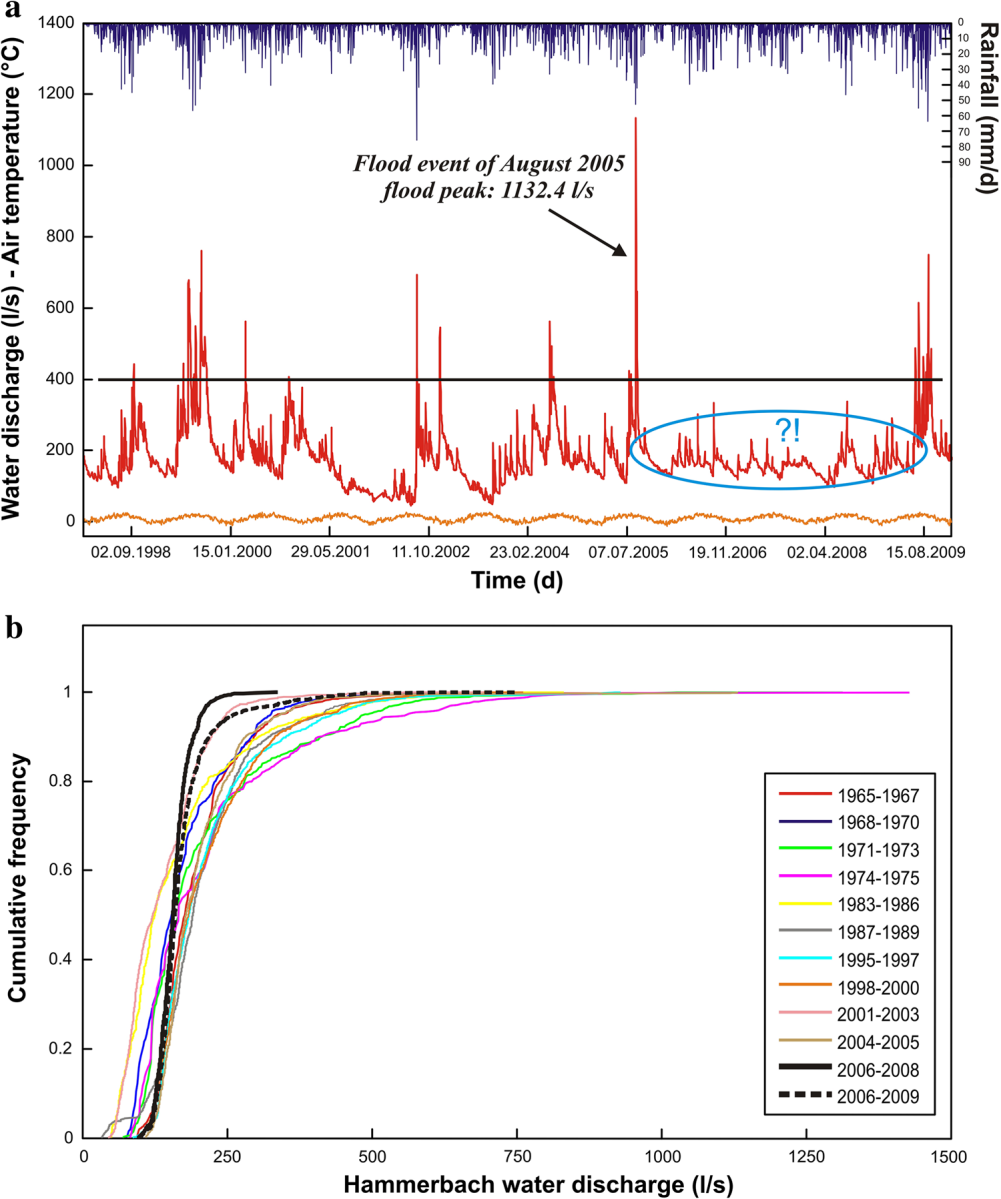
Hammerbach spring data. **a** Hammerbach daily discharge (*red line*), precipitation (*blue bars*) and air temperature (*orange line*) data from 1998 to 2010. *Black line* discharge of 400 l/s, *blue circle* changed behaviour from August 2005 to June 2009. Precipitation data are the computed average of the six stations located in the region; the temperature data are taken from a station located on the western side of the Mur valley. **b** Cumulative frequency curves of the Hammerbach discharge from 1965 to 2010. The *bold solid black line* represents the changed behaviour between 2006 and end 2008, whereas the *bold dashed black curve* represents the period from 2006 to 2009 including the return to the previous drainage behaviour of the Hammerbach spring

The Hammerbach spring hydrograph between 1998 and 2010 (Fig. 2a) exhibits a changed behaviour after a major flood event in August 2005. From then onward, the spring response appears to be more damped and the peak discharge did not exceed 400 l/s over a period of about 4 years. Moreover, the baseflow appears to have increased. In contrast, precipitation appeared to be rather unchanged. Unfortunately, there is not enough discharge data of the Schmelzbach spring to identify potential changes in its discharge behaviour. Since June 2009, several floods due to intense storm events indicate that the Hammerbach has recovered to its previous flashy behaviour.

When plotting cumulative frequency curves of 3-year periods of the Hammerbach discharge from 1965 to 2010 (Fig. 2b), the curve from 2006 to 2008 (the bold solid black line) reveals a rather damped discharge behaviour with a lower maximum and a higher minimum compared to the other curves. If the year 2009 is included in the curve from the previous 3 years, the resulting bold dashed black curve is more similar to the curves prior to 2005. This observation confirms that the hydrological behaviour of the Hammerbach spring from 2006 to 2008 differs from that of the years before and after that time period.

**Fig. 3:**
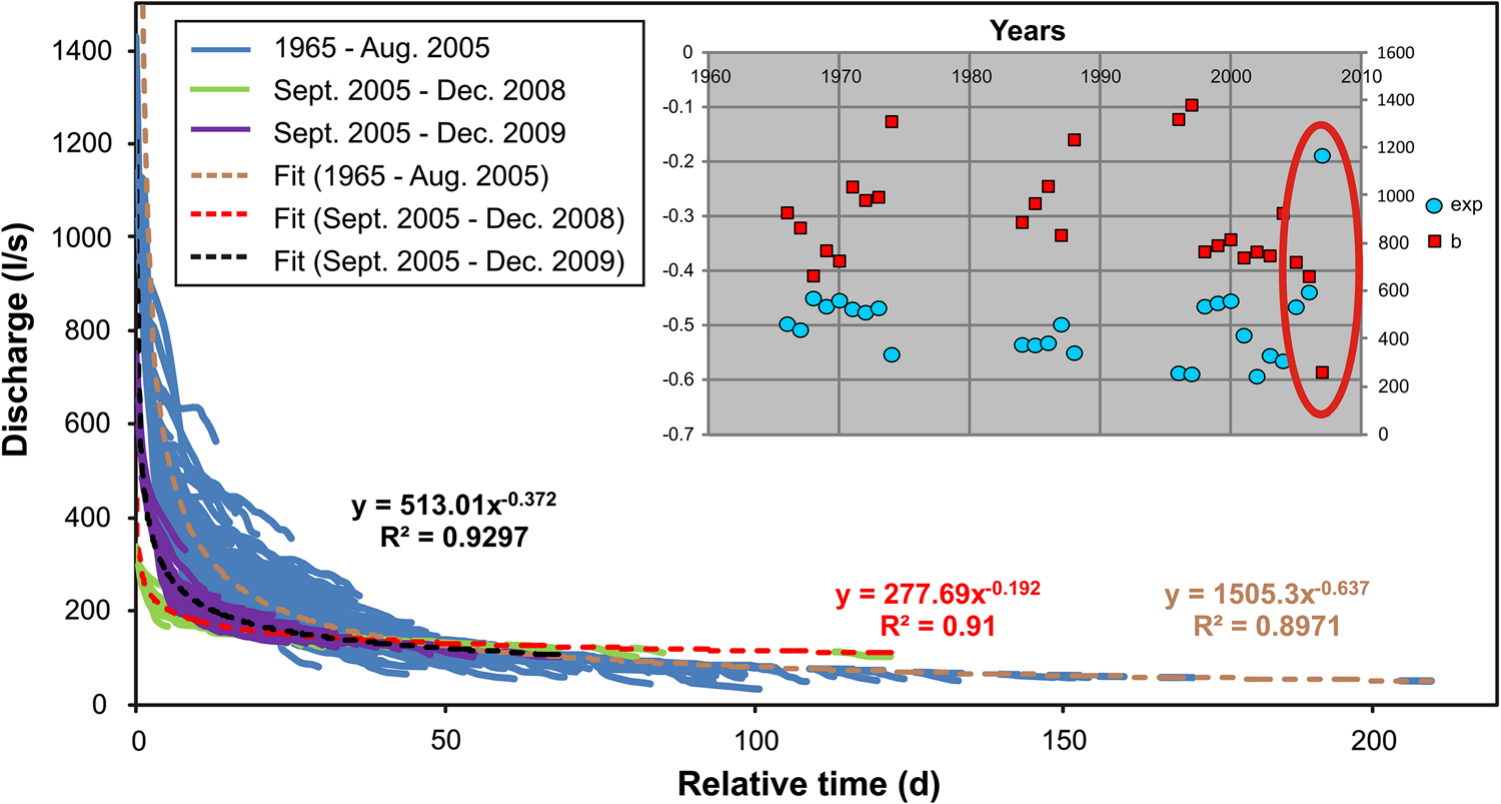
Master recession curves of the Hammerbach between 1965 and 2010. The changed recession behaviour within the period from 2005 to 2009 is evident. *Inset* coefficient and exponent of a power law fit of the master recession curves for time periods of three years. The change after 2005 is highlighted by the jump of the constant and exponent. Master recession curves were computed with the method according to Posavec et al. (2006)

The Hammerbach master recession curves shown in Fig. 3 confirm the hypothesis of a different hydrological behaviour between August 2005 and June 2009. The recession behaviour of the period 1965–2005 is clearly different from that of the period 2005–2008. The return to the pre-2005 behaviour is illustrated by the violet curve from 2005 to 2009, which is closer to the recession curves from 1965 to 2005. The same result was found when master recession curves of 3-year periods between 1965 and 2005 were compared to that of the period 2005–2008. This is shown in the inset of Fig. 3, where a similar trend is observable when looking at the exponent and the coefficient of a power law fit of the master recession curves of 3-year periods before 2005, whereas the period between 2006 and 2009 (surrounded by the red circle) is clearly different. As master recessions curves provide information about the aquifer structure (Kresic and Bonacci 2010, p. 134), these results suggest that the observed hydrological change is caused by changes within the binary karst catchment rather than by varying meteorological conditions. Since there are no evident significant changes in land use, deforestation and/or housing, which may potentially have influenced the flow regime of the Lurbach stream in the upper part of the catchment, it is an obvious idea to assume that changes occurred within the karst aquifer of the Tanneben massif. Although a sediment barrage in the Lurbach stream is dredged from time to time where some sinkholes are reported, it appears unlikely that this may have caused a change in the hydrological behaviour over a period of 4 years.

A possible explanation of the observed hydrological change is a change in the karst drainage system due to the flood event of August 2005. Potentially an aggradation of sediments in the Hammerbach conduit network caused a decrease of the hydraulic conductivity and/or changes in the storage properties. This is a plausible process, as sediment redistributions are common in karst aquifers (e.g. Farrant and Smart 2011) and are noticed regularly in the accessible parts of the cave system after stronger rainfall events (Kübeck et al. 2013). The apparent return to the former behaviour may be explained by the re-excavation of the sediments previously plugging conduit sections during the major storm events of summer 2009. Results from a tracer experiment in December 2008 (Oswald 2009) further indicate that the above-mentioned overflow from the Hammerbach system to the Schmelzbach may occur at a lower Hammerbach discharge than earlier reported by Harum and Stadler (1992). In addition, the transit times for this particular tracer experiment were almost 60 h (Oswald 2009), whereas tracer experiments conducted prior to August 2005 show transit times of less than 40 h at comparable discharge rates (Behrens et al. 1992). This too might be explained by the plugging of infiltration sinkholes and flow paths by sediments or tree lumps, caused by the major flood event of August 2005.

## Modelling

Karst aquifers are known for their large but organized heterogeneities, which can be conceptualized as a dual flow system consisting of a highly conductive network of solution conduits that is embedded in the less conductive fissured carbonate rock (e.g. Kiraly 1998). These dual flow characteristics are responsible for intrinsic difficulties in modelling groundwater flow in karst terrains. An overview of modelling approaches that have been proposed to overcome these difficulties is provided by Rehrl and Birk (2010). One approach frequently employed for generic investigations into flow and transport processes in karst aquifers are hybrid models, which couple a pipe flow model representing the network of solution conduits to a continuum model representing the fissured rock (Teutsch and Sauter 1991). Applying hybrid models to real karst aquifers, however, is highly challenging, since adequate information about the geometric and hydraulic properties of the conduit system are rarely available. Therefore, single-continuum groundwater flow models such as MODFLOW (Harbaugh et al. 2000) are frequently employed for these purposes (e.g. Worthington 2009; Ravbar et al. 2011). The flow calculation in this type of model typically is based on Darcy’s law, thus assuming only laminar flow conditions. Reimann et al. (2011) demonstrated that spring hydrographs simulated with MODFLOW may differ significantly from those obtained with a hybrid model that accounts for turbulent flow in solution conduits. Yet results obtained with an extension to MODFLOW that accounts for turbulent flow were found to be in agreement with those from the hybrid model. In this work MODFLOW is employed considering first only laminar flow. Subsequently, it is attempted to apply a recently developed turbulent-flow package for MODFLOW (Shoemaker et al. 2008).

### Laminar flow

**Fig. 4:**
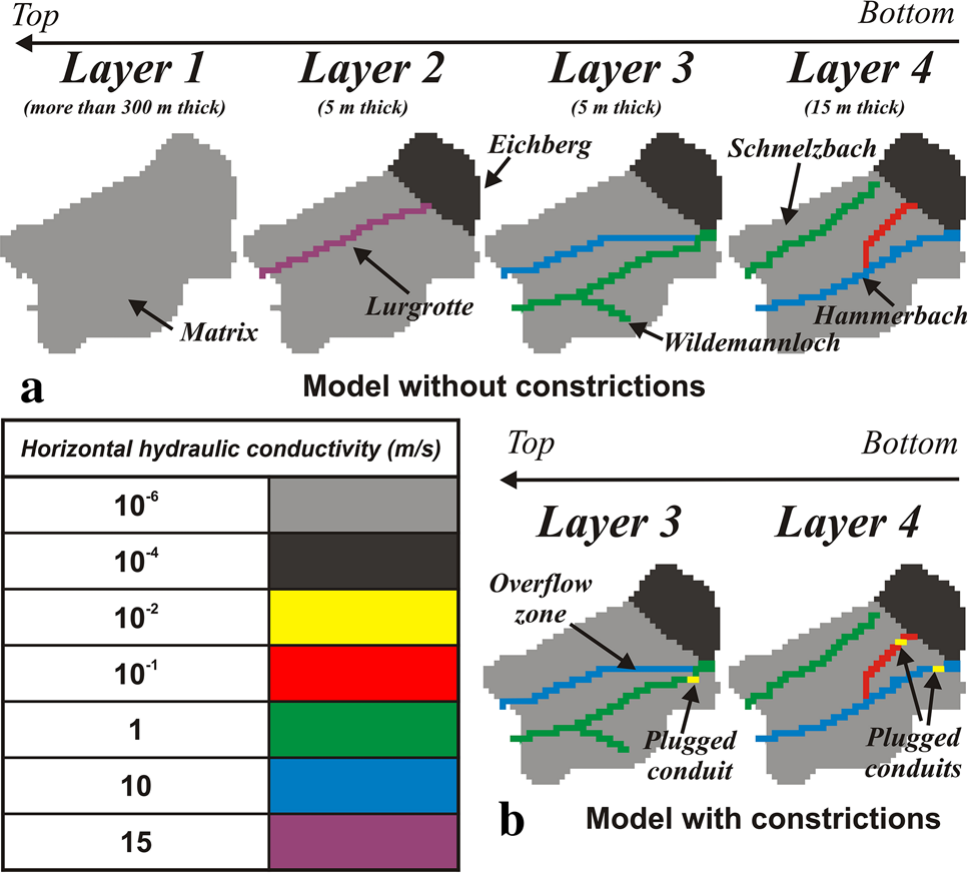
Model setup. Comparison of the horizontal hydraulic conductivities and the geometry between the model without constrictions (**a**) and the model with constrictions (**b**). The plugged conduits are represented in *yellow* in **b**. With these two model setups it is attempted to reproduce the pre-event (**a**) and post-event (**b**) drainage behaviour of the Lurbach system. The specific storage of the matrix was set to 10^−5^ m^−1^ for the two models, whereas it was given 0 in the conduit for the unplugged case and 0.5 m^−1^ to the constrictions of the plugged case. The vertical conductivity was set to 10^−4^ m/s for the whole model range, except where the Lurbach allogenic input is added, where a high value of 10 m/s was defined in order to simulate the sinkholes

The design of the numerical model is based on the conceptual understanding of the Lurbach system described above. The model is a simplified Lurbach system representing only the autogenic sub-catchment and consists of four layers that differ in thickness and horizontal hydraulic conductivity (Fig. 4a) to capture the complexity of the multi-level cave system. The catchment is made up of a low conductivity matrix (light grey and dark grey in Fig. 4), whereas the highly conductive flow paths introduced in the layer 2–4 represent the Lurgrotte cave and the assumed Hammerbach conduit system. The structure of the conduit system was designed to account for the behaviour of the Lurbach system described in Harum and Stadler (1992): during low water conditions the lower layer (layer 4) drains most of the allogenic recharge and both Hammerbach and Schmelzbach systems are separated. When the allogenic input rises above the drainage capacity of the layer 4, the layer 3 begins to drain and the overflow towards the Schmelzbach aquifer is activated. The layer 2 becomes prominent during strong storm events and allows water to flow through the upper levels of the Lurgrotte cave system (see Kübeck et al. 2013). The uppermost layer represents the more than 300 m thick karst matrix of the Tanneben massif which might serve as further storage component if extreme floods are considered (high historical floods reported in Benischke et al. (1994) indicate that part of the Semriach basin was flooded in 1812 and 1827).

Three steady-state model scenarios were designed using autogenic recharge based on the formula of Turc (e.g. Gray 1970), the minimum, mean and maximum annual precipitation depths, respectively, and a mean annual air temperature (7.4 °C) reported in Harum and Stadler (1992). Correspondingly, the lowest, mean and highest Lurbach discharges reported by Harum and Stadler (1992) were used as concentrated allogenic recharge. The allogenic recharge was given as localised input where the Hammerbach and Schmelzbach conduits begin (at the border between the Eichberg zone and the matrix zone in Fig. 4) and along the Lurbach riverbed with emphasis on the conduits draining the Hammerbach system. The autogenic recharge was given as a constant flux rate (in m/s) within the entire model domain. The three steady-state models were calibrated to the lowest, mean and highest discharges at the Hammerbach spring and Schmelzbach outlet by adjusting the hydraulic conductivity of the low-permeability matrix and the highly conductive flow paths. These simulations gave reasonable results (Mayaud 2010) and confidence into the model setup, especially concerning the aforementioned intercatchment flow and its dependence on the hydrological conditions. The resulting model was subsequently employed for transient simulations to reproduce the Hammerbach spring hydrograph of the flood event of August 2005 (Fig. 2a). This event was chosen because of its nearly undisturbed long-term recession, because the discharge peak reached almost the Hammerbach filling capacity, and because it is likely the last strong event prior to the change in the system behaviour. Unfortunately no Lurbach data are available for this period. Thus, only the autogenic recharge was computed using daily precipitation data minus 50 % evaporation rate, whereas the concentrated allogenic recharge component was adjusted in the model calibration. For the transient simulations the specific yield of the matrix was set to 0.01 in the whole unconfined aquifer, whereas the specific storage was defined as 0 in the conduits and 10^−5^ m^−1^ in the matrix.

**Fig. 5:**
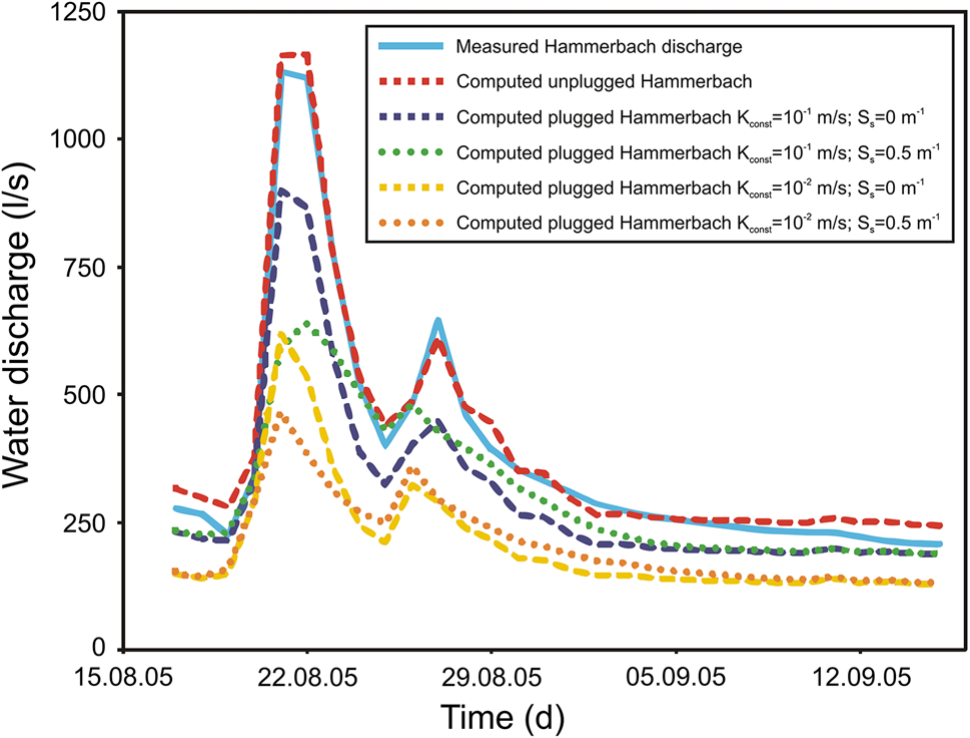
Measured and simulated Hammerbach discharge for the flood event of August 2005 without and with constrictions and sediment storage in the conduit network (see the location of constrictions in Fig. 4b). The *violet curve* represents the discharge when constrictions with hydraulic conductivity of 10^−1^ m/s are assigned, the *yellow curve* when constrictions with hydraulic conductivity of 10^−2^ m/s are assigned. *The green and orange curves* represent the response when a high specific storage value of 0.5 m^−1^ (compared to the value of 0 given for the previous cases) is given to the constrictions

Figure 5 shows the simulated (dashed red line) compared to the measured (solid blue line) discharge of the Hammerbach. The simulation matches the observed discharge behaviour reasonably well and makes the assumptions of the model setup a plausible option (admittedly not the only one). To examine potential effects of sediment aggradations that may have caused the plugging of conduits within the Hammerbach system, the hydraulic conductivity of some conduit cells was lowered (see Fig. 4b). The resulting flow constrictions cause an increase of the water table and of the hydraulic gradient within the conduits, thus forcing more water to flow towards the Schmelzbach network. As a consequence, the Hammerbach peak discharge is lower in these scenarios compared to the scenario without constriction (Fig. 5). This is qualitatively similar to the behaviour of the Hammerbach spring hydrograph after the flood event of August 2005 (Fig. 2a). It is evident that the constricted conduit sections prevent a part of the Lurbach water from flowing immediately towards the Hammerbach spring. Thus, the water flows more frequently towards the Schmelzbach drainage network and explains the damped discharge behaviour presented in the previous paragraphs. This is in accordance with a tracer experiment in 2008 where overflow to the Schmelzbach system was already observed at ~135 l/s (Oswald 2009) compared to the previously reported threshold of ~200 l/s (Behrens et al. 1992).

However, the observed increase in the baseflow is not reproduced by the simple assumption of constrictions with lower hydraulic conductivity. A potential explanation to this increase is that the aggradation of the sediments may not only have changed the hydraulic conductivity but also the storage properties of the Hammerbach aquifer. To examine if changes of the storage properties related to the sediments in the constrictions may have caused the increase of the baseflow observed after the major flood event in 2005, a simple hypothesis was considered by assigning high values of storativity to the conduit constriction (storativity was set to zero before). Results are presented in Fig. 5 (dotted green and orange lines) and show that the baseflow of the Hammerbach is increased if the storativity in the constriction is increased. Then, the peak flow is more damped compared to the scenarios without change in the storativity. Thus changes in the storativity might account for the second observed characteristic of the Hammerbach behaviour between August 2005 and June 2009.

### Turbulent flow

**Fig. 6:**
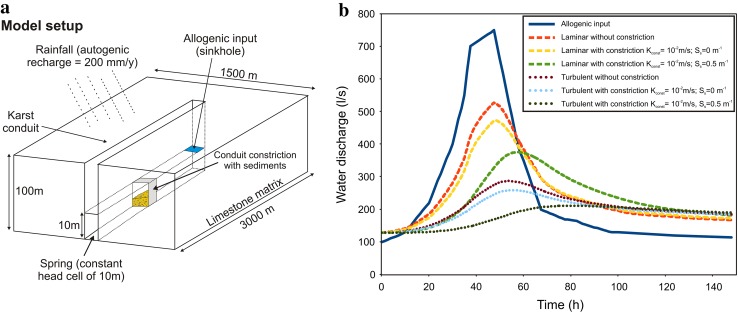
Comparison of laminar and turbulent flow conditions of the simplified model setup. **a** Model setup including the geometrical assumptions and the boundary conditions. **b** Changes in spring discharge considering and neglecting turbulent flow conditions within the conduit. Moreover, the influence of constrictions within the conduit is considered by changing conductivity and specific storage of the constriction. The *red*, *yellow* and *green dashed curves* are the responses neglecting turbulent flow, whereas the *dotted purple*, *light-blue* and *dark-green curves* represent the spring responses taking turbulent flow into consideration

As flow in such a mature karst aquifer is likely to be locally (i.e. in the karst conduits) turbulent, it was an obvious objective to integrate turbulent flow into the modelling approach. The program used for this purpose was the Conduit Flow Process (CFP) for MODFLOW developed by Shoemaker et al. (2008). Only a simplified one-layer model was realized with CFP due to convergence problems; more complex multi-level models are subject of ongoing modelling attempts. The geometry of the model aquifer had to remain simple and comprised only a square catchment with one conduit representing the Hammerbach system (see Fig. 6a). The spring response to an artificial event was computed taking into account turbulent flow and compared with a standard MODFLOW approach (laminar flow only). The results (Fig. 6b) show an agreement with the previous results of Fig. 5 with an increase of the baseflow correlated to an increase of the storativity in the constrictions. It is important to note that the damping is much stronger in the model scenarios that account for turbulent conduit flow than in those ignoring turbulent flow, which is in accordance with the findings by Reimann et al. (2011). These authors showed that the hydraulic gradient in the conduits is higher if turbulent flow is taken into account. As a result, the conduit hydraulic heads are higher in the model accounting for turbulent flow than in a purely laminar flow model. Thus, flow from the matrix to the conduit is more strongly reduced in the turbulent flow model, which causes an increase of storage in the fissured matrix that is not considered in the laminar model. Therefore, turbulent flow needs to be considered to be able to quantitatively assess storage effects in such a karst system.

## Discussion

A change of hydrological behaviour was observed at the Hammerbach spring during a period of nearly 4 years. This change resulted in a damped behaviour of the peak flow combined with an increase in the baseflow. The analysis of discharge data using cumulative frequency and master recession curves combined with previously reported tracer experiment results suggest that the change is caused by processes within the karst system rather than by climatic and/or anthropogenic factors. Based on field investigations it is suggested that sediments may constrict and/or plug karstic conduits and lead to the damped discharge behaviour observed at the spring. A simplified distributive groundwater model was built based on the current conceptual understanding of the system in order to investigate if conduit constrictions and sediment plugging in karstic conduits may cause changes in the spring response similar to those identified from the field observations. The simulation results are in accordance with the aforementioned hypotheses: the modification of the hydraulic conductivity and the specific storage in the karstic conduit led to a more damped discharge at the spring.

It should be noted, however, that the design and calibration of the groundwater model is highly non-unique because of the scarcity of data. Thus, the model should be viewed as an interpretative (Anderson and Woessner 1992, p. 4) model aimed at improving the conceptual understanding of the hydrogeological system at the field site, but not as a predictive model. For the latter purpose, a parsimonious lumped-parameter rainfall-runoff model appears to be more appropriate. When applying such a model to simulate the Hammerbach spring Wagner et al. (2013) found that it was unable to reproduce the observed change in the discharge behaviour with parameter sets identified from other time periods. Within the 4-year period that is characterized by the damped discharge behaviour a reasonable model fit was obtained only by an increased overflow towards the neighbouring sub-catchment of the Schmelzbach aquifer and an increased storage capacity in the aquifer itself. This is in agreement with the findings from the data analysis and groundwater modelling presented here, which suggest that changes of hydraulic properties rather than climatic factors are responsible for the observed hydrological change.

## Conclusions

Data analysis reveals a change in the hydrological behaviour of the Lurbach system after a storm event in August 2005 until June 2009. The spring response appears to be more damped and the baseflow higher than before, which is probably related to the aggradation of sediments in conduit sections during the storm event in 2005. Using the distributive groundwater flow model MODFLOW the changed behaviour of the Hammerbach spring was qualitatively reproduced by incorporating sections of low conductivity and high storage in highly conductive flow paths representing the conduit network (to mimic plugged conduit sections). Thus, the single-continuum model MODFLOW was found to be able to reproduce the transient behaviour observed at the spring. This model, however, does not account for turbulent flow in karst conduits. A MODFLOW package that considers turbulent flow in the continuum model (CFP) was employed to assess potential effects of turbulence on the transient flow behaviour. Results from a highly simplified model scenario demonstrate that the storage in the fissured matrix is underestimated if turbulent flow is ignored. The implementation of turbulent flow in a more realistic model setting is the subject of future work.
